# Protective Role of Axial Length in Diabetic Retinopathy: A Case-Control Study

**DOI:** 10.7759/cureus.96178

**Published:** 2025-11-05

**Authors:** Aikaterini E Mouzaka, Aristeidis Chandrinos, Irini Chatziralli, Eleni Chatzichristou, Themistoklis K Gialelis

**Affiliations:** 1 Optics and Optometry Division, Investigative Techniques in Optometry Research Group, Department of Biomedical Sciences, University of West Attica, Athens, GRC; 2 2nd Department of Ophthalmology, National and Kapodistrian University of Athens School of Medicine, Athens, GRC

**Keywords:** axial length, diabetes mellitus, diabetic retinopathy, myopia, ocular microvasculature

## Abstract

Background

Diabetic retinopathy (DR) is the most common microvascular complication of type 2 diabetes mellitus (T2D) and a leading cause of visual impairment. Axial length (AL) of the eye has been implicated in retinal structure and may influence DR pathogenesis. This study aimed to test the hypothesis that longer ocular AL exerts a protective effect against the development and progression of DR. In addition, it sought to examine secondary associations between AL and demographic or clinical variables, including sex, diabetes duration, insulin use, and presence of diabetic macular edema.

Methods

A total of 455 adults aged 50-85 years were included, of whom 135 (29.7%) had confirmed T2D. AL was measured using the IOL Master 500, and DR staging was performed through indirect ophthalmoscopy and optical coherence tomography (OCT). Due to non-normal data distribution, non-parametric tests were used (Mann-Whitney U, Kruskal-Wallis, Spearman, Pearson Chi-Square).

Results

Diabetic patients exhibited significantly lower median AL compared to non-diabetics (23.26 mm vs. 23.50 mm, *p* = 0.006). Among diabetics, males had longer AL than females (23.37 mm vs. 22.86 mm, *p* = 0.001). Participants without any signs of DR had significantly greater AL than those with any DR stage (23.63 mm vs. 23.00 mm, *p* = 0.042). Furthermore, AL varied significantly across DR stages (*p* = 0.017), with a trend toward shorter length in more advanced stages.

Conclusions

Longer AL is inversely associated with both the presence and severity of DR. AL may represent a protective anatomical factor and could be considered in the clinical evaluation and risk stratification of patients with T2D.

## Introduction

Diabetes mellitus (DM) is a prevalent chronic metabolic disorder characterized by hyperglycemia resulting from impaired insulin secretion, insulin action, or both. Type II diabetes mellitus (T2DM), which constitutes the majority of diabetes cases worldwide, is particularly common in older adults and is closely associated with obesity, sedentary lifestyle, and genetic predisposition [1,2].

The global burden of diabetes is substantial, with over 537 million individuals affected in 2021 and projections estimating a rise to 783 million by 2045 [3]. Beyond its systemic complications, T2DM is a major contributor to ocular morbidity, with diabetic retinopathy (DR) representing one of its most serious and sight-threatening microvascular complications [4].

DR arises from chronic hyperglycemia-induced damage to the retinal microvasculature, leading to capillary basement membrane thickening, pericyte loss, endothelial dysfunction, and ultimately to increased vascular permeability and capillary occlusion [5]. The pathophysiological progression of DR is typically divided into two major stages: non-proliferative diabetic retinopathy (NPDR), which is characterized by microaneurysms, dot-blot hemorrhages, and retinal edema, and proliferative diabetic retinopathy (PDR), which involves neovascularization as a response to retinal ischemia and elevated levels of angiogenic factors such as vascular endothelial growth factor (VEGF) [6]. DR is a leading cause of vision impairment and legal blindness among working-age adults in developed countries, with significant implications for patient quality of life and healthcare systems [7].

The ocular axial length (AL) - defined as the distance from the anterior corneal surface to the retinal pigment epithelium - is a fundamental biometric parameter of the eye, with significant implications in refractive error classification and intraocular lens calculation [8].

In recent years, increasing interest has been directed toward understanding whether AL may also play a role in the pathogenesis or severity of DR. Several studies have hypothesized that longer ALs may exert a protective effect against the development of DR, possibly due to mechanical stretching of the retina that reduces capillary density or due to alterations in ocular blood flow [9-10]. Conversely, shorter axial lengths have been associated with increased risk of DR, possibly due to higher retinal metabolic demands and vascular density per unit area [11].

Despite these hypotheses, the existing literature on the relationship between AL and DR is limited and presents contradictory findings. The present study primarily aimed to evaluate whether longer ocular axial length is associated with a lower prevalence and severity of DR, based on the hypothesis that increased AL may exert a protective anatomical effect. Secondary objectives included examining the relationship between AL and key demographic and clinical factors - such as age, sex, duration of diabetes, insulin therapy, and diabetic macular edema - in order to provide a more comprehensive understanding of factors influencing biometric ocular characteristics in patients with T2D. By employing a robust case-control design with standardized biometric measurements and ophthalmoscopic classification of DR, this study seeks to contribute valuable evidence to a poorly understood area of diabetic eye disease and potentially inform future screening or risk stratification strategies.

## Materials and methods

Study design

This study was designed as a case-control investigation aimed at evaluating the AL of the eye in adults with T2D and examining its potential association with the presence and severity of DR. The research was conducted at the 2nd University Ophthalmology Department of the National and Kapodistrian University of Athens, located within the University General Hospital “Attikon”, in collaboration with the Department of Biomedical Sciences, University of West Attica.

The study was conducted between January 2021 and December 2024. Ethical approval for the study was granted by the Institutional Review Board of the University of West Attica (Approval No. 100475/09-12-2020) during the 22nd meeting of the Ethics Committee on 14 December 2020.

The diagnosis of T2DM was based on established clinical criteria in accordance with World Health Organization and American Diabetes Association guidelines. Specifically, participants were classified as diabetic if they had an HbA1c level ≥ 6.5% (48 mmol/mol), fasting plasma glucose ≥ 126 mg/dL (7.0 mmol/L), or a documented diagnosis of T2D with current antidiabetic treatment (oral hypoglycemic agents and/or insulin).

Inclusion and exclusion criteria

The study population comprised two groups of adult participants: one group of individuals diagnosed with T2D and a matched control group of non-diabetic individuals. Eligible participants in both groups were between 50 and 85 years of age. Patients diagnosed with type I DM, those under 50 years of age, or individuals with a history of strabismus were excluded from the study to ensure homogeneity of ocular parameters and to eliminate confounding ocular conditions. In addition, participants with other ocular pathologies that could influence ocular biometry or the retinal microvasculature, such as glaucoma, uveitis or other inflammatory eye diseases, retinal vascular occlusions, or a history of significant ocular trauma, were also excluded. Subjects with co-existing systemic hypertension were not excluded but were documented, as this variable may act as a potential confounder in DR progression and was considered in the interpretation of results.

AL measurements and DR staging were performed per eye, but for statistical analysis, only one eye per participant (the right eye) was included to avoid inter-eye correlation effects, given that DR staging may differ between eyes.

Ophthalmological evaluation

All participants underwent a comprehensive ophthalmological evaluation during their scheduled visits for either routine eye examination or cataract surgery pre-assessment. The examination included detailed documentation of demographic characteristics, medical history with particular emphasis on diabetes duration and glycemic control, as well as a thorough ocular history including previous diagnoses, treatments, and refractive errors. This initial evaluation was performed by an experienced ophthalmologist who was not the treating physician of any of the enrolled patients, thereby ensuring the independence of the assessment process.

Diagnosis and Staging of Diabetic Retinopathy

The diagnosis and staging of DR were conducted using indirect ophthalmoscopy (fundoscopy) following pupil dilation. Fundoscopic evaluation allowed for the classification of DR according to internationally accepted clinical criteria, distinguishing between non-proliferative and proliferative stages based on the presence of microaneurysms, hemorrhages, cotton wool spots, venous beading, intraretinal microvascular abnormalities (IRMA), and neovascularization.

The diagnosis and grading of DR were performed according to the International Council of Ophthalmology Global Diabetic Retinopathy Severity Scale, which aligns with the Early Treatment Diabetic Retinopathy Study (ETDRS) classification. DR was categorized as no DR, mild/moderate/severe non-proliferative DR, or proliferative DR based on standard ophthalmoscopic findings.

Measurement of Axial Length

Ocular AL was measured using the IOL Master 500 (Carl Zeiss Meditec AG, Germany), a non-contact optical biometry device based on the principle of partial coherence interferometry (PCI). This method allows for precise measurements of the distance from the anterior surface of the cornea to the retinal pigment epithelium using infrared light at a wavelength of approximately 780 nm. The measurement is based on the detection of time delays between light reflections from different intraocular structures, which are then processed through an interferometric algorithm to derive an accurate estimate of AL.

Measurement Protocol and Quality Control

Each measurement session involved five repeated scans, with only values showing a signal-to-noise ratio (SNR) greater than 2.0 being considered valid. The final AL for each eye was calculated as the mean of these five values. The high degree of repeatability and precision of the IOL Master, with an accuracy of approximately ±0.02 mm, renders it a reliable tool for biometric assessments in both clinical and research contexts. Additionally, its non-contact nature eliminates the need for corneal anesthesia and minimizes the risk of contamination or infection.

Ethical considerations and data anonymization

Prior to each measurement, the contact surfaces of the biometry device (chin and forehead rests) were disinfected with 70% ethanol to ensure hygienic conditions. Only anonymized, coded data were used for analysis. The identifiers retained included a numeric code, year of birth, sex, and diabetic status. All procedures were performed in accordance with the principles outlined in the Declaration of Helsinki, and written informed consent was obtained from all participants prior to enrollment.

Statistical analysis

Statistical analysis was performed using IBM SPSS Statistics version 26.0 (IBM Corp., Armonk, NY, USA). The level of statistical significance was set at p < 0.05. The distribution of all variables was assessed for normality using the Kolmogorov-Smirnov and Shapiro-Wilk tests. Due to deviations from normality, non-parametric statistical tests were applied for independent group comparisons.

Specifically, the Mann-Whitney U test was used to compare two independent groups (e.g., diabetic vs. non-diabetic individuals, males vs. females), while Spearman’s rank correlation coefficient (rho) was employed to examine associations between continuous, non-normally distributed variables (e.g., AL and age or diabetes duration). The Kruskal-Wallis test was used for comparisons involving more than two independent groups (e.g., DR stages). Finally, associations between categorical variables (e.g., sex and diabetes status) were assessed using Pearson’s Chi-Square test.

## Results

Demographic characteristics

A total of 455 individuals were included in the study, of whom 320 (70.3%) were non-diabetic and 135 (29.7%) had a confirmed diagnosis of T2D. The mean HbA1c level among diabetic participants was 7.4 ± 1.2%. Participant age ranged from 50 to 85 years, with an overall range of 58 years. The median age of the sample was 73 years. Of all participants, 255 (56.0%) were male and 200 (44.0%) were female. The mean age among non-diabetic participants was 72.31 ± 9.48 years, while for diabetic participants it was 71.49 ± 8.04 years. The median ages were 73 and 70 years, respectively. Age range in the non-diabetic group was 50-83 years, while in the diabetic group it was 50-85 years.

The comparison of age between the two groups was performed using the non-parametric Mann-Whitney U test due to deviation from normal distribution. The difference was not statistically significant (p = 0.226), suggesting that the two groups were comparable in terms of age.

Regarding gender distribution, the non-diabetic group included 184 males (57.5%) and 136 females (42.5%), while the diabetic group consisted of 69 males (51.1%) and 66 females (48.9%). The association between gender and diabetes status was assessed using Pearson’s Chi-Square test (Χ² (1) = 1.751), which showed no statistically significant difference (p = 0.210). Thus, there were no systematic differences in gender distribution between the two groups.

Comparison of axial length between diabetic and non-diabetic participants

The mean AL among non-diabetic individuals was 23.66 ± 1.16 mm (median: 23.50 mm; range: 21.34-29.95 mm), while among diabetic individuals it was 23.25 ± 2.32 mm (median: 23.26 mm; range: 0.00-30.42 mm). The difference in AL between the two groups was statistically significant (p = 0.006), indicating that diabetic participants had significantly shorter AL compared to non-diabetics.

Table 1 summarizes the comparison of key demographic and biometric characteristics between diabetic and non-diabetic participants.

**Table 1:** Comparison of Characteristics Between Diabetic and Non-Diabetic Participants Data for continuous variables are expressed as median (Minimum – Maximum) and for categorical variables as N (%). Group comparisons for continuous variables were performed using the Mann-Whitney U test, and for categorical variables using the Chi-square test. Test statistics are reported as U for Mann-Whitney U and χ² for Chi-square. Statistical significance was set at p < 0.05.

Variable	Non-Diabetic (n = 320, 70.3%)	Diabetic (n = 135, 29.7%)	Test statistic	p-value
Age (years)	Median: 73 Range: 50-83	Median: 70 Range: 50-85	U = 19584.0	0.226
Sex	Men: 184 (57.5%) Women: 136 (42.5%)	Men: 69 (51.1%) Women: 66 (48.9%)	χ² = 1.58	0.210
Axial Length (mm)	Median: 23.50, Range: 21.34–29.95	Median: 23.26, Range: 0.00–30.42	U = 17456.5	0.006

Factors associated with axial length in diabetic patients

In the subgroup of patients with T2D, associations between AL and demographic or clinical characteristics were explored, including sex, age, diabetes duration, insulin use, diabetic macular edema, and retinopathy stage. A statistically significant difference in AL was observed between sexes, with male patients demonstrating greater AL values than females (p < 0.001). Spearman’s rank correlation coefficient revealed no significant association between AL and age (rho = -0.079, p = 0.362) or diabetes duration (rho = -0.012, p = 0.918).

Comparison of AL based on insulin therapy did not yield statistically significant results (p = 0.102), although a trend toward shorter AL in insulin-treated patients was noted. Similarly, the presence of diabetic macular edema did not significantly influence AL value (p = 0.333).

Analysis of AL across DR stages using the Kruskal-Wallis test revealed a statistically significant difference (p = 0.017). Specifically, higher AL values were observed in earlier stages of DR, while more advanced stages were associated with progressively shorter AL. These findings suggest a possible inverse relationship between AL and DR severity in diabetic patients.

Furthermore, a binary comparison was performed between diabetic patients with no signs of DR (stage 0) and those with any level of retinopathy (stages 1 to 4). The Mann-Whitney U test demonstrated a statistically significant difference (p = 0.042). Patients without DR had significantly longer AL values compared to those with DR, potentially indicating a protective role of greater axial length against the development of DR.

Table 2 summarizes the comparison of median AL values and measurement ranges across clinical and demographic categories.

**Table 2:** Comparison of Median Axial Length (AL) and Value Range Across Clinical and Demographic Categories Data are expressed as Median (Minimum – Maximum). Mann-Whitney U test was applied for two-group comparisons (Sex, Insulin Use, Macular Edema, DR Group) and Kruskal-Wallis test for multiple groups (DR Stage). Test statistics are reported as U and H values, respectively. Statistical significance was set at p < 0.05.

Category	Median AL (mm)	Minimum – Maximum (mm)	Test Statistic (T/χ²/F)	p-value
Sex	-	-	t = 3.35	0.001
Female	22.86	21.93 – 28.52	-	-
Male	23.37	22.08 – 30.42	-	-
Insulin Use	-	-	t = 1.65	0.102
No	23.30	22.04 – 25.73	-	-
Yes	23.01	21.93 – 30.42	-	-
Macular Edema	-	-	t = 0.98	0.333
Absent	23.22	21.93 – 30.42	-	-
Present	23.94	22.61 – 28.52	-	-
DR Stage	-	-	F = 3.27	0.017
0	23.63	23.13 – 24.12	-	-
1	23.07	22.77 – 30.42	-	-
2	23.22	22.08 – 28.52	-	-
3	23.26	22.29 – 24.58	-	-
4	22.56	0.00 – 25.73	-	-
DR Group	-	-	t = 2.08	0.042
No DR	23.63	23.13 – 24.12	-	-
With DR	23.00	21.80 – 25.40	-	-

## Discussion

This study was the first in Greece to investigate the association between AL and the presence of T2D, as well as its relationship with specific clinical characteristics within the diabetic patient group. It was found that diabetic patients exhibited significantly shorter AL values compared to non-diabetic individuals. Within the diabetic group, AL differed significantly by sex, with males demonstrating higher values than females. In contrast, no statistically significant differences were observed with respect to age, duration of diabetes, insulin use, or the presence of diabetic macular edema. However, a significant association was identified between AL and DR, with patients without signs of DR showing longer AL compared to those with clinically evident retinopathy. Additionally, statistically significant differences in AL were observed across different stages of DR. These findings support the literature-based hypothesis that axial length may serve as a biometric marker with a potentially protective effect against the development or progression of DR, while also highlighting the influence of sex on this anatomical parameter.

The inverse relationship between AL and the risk of DR has been well documented in the literature. Numerous studies have demonstrated that increased AL may confer a protective effect against the onset and progression of DR, potentially through structural, metabolic, and inflammatory mechanisms. Early evidence by Baker et al. and more recent data by Thakur et al. showed that high myopia significantly reduced the odds of DR, especially among high-risk individuals with specific HLA phenotypes [12,13].

Subsequent studies have reinforced this inverse association. Klein et al. and Wang et al. reported that each millimeter increase in AL was associated with a decreased risk of DR [14,15]. Wang et al. quantified this effect with an odds ratio (OR) of 0.81 per mm increase in AL, even after adjustment for confounding factors [15]. Xu et al. similarly reported a protective association with an OR of 0.48 [16]. Conversely, Varma et al. and Xie et al. observed that shorter AL, suggestive of hyperopia, may be linked to higher levels of VEGF and other pro-inflammatory cytokines, thereby increasing DR risk [17,18].

Population-based studies further support these findings. Yang et al. and Pan et al. noted a significant decline in DR incidence with increasing AL [10,19]. Notably, Ten et al. showed a 56% risk reduction associated with high myopia (OR 0.44), while Moss et al. and Chao et al. emphasized the protective role of longer AL in younger-onset diabetes cohorts [20-22].

Mechanistically, larger AL may reduce retinal hypoxia and metabolic demand, as proposed by Lim et al. and validated by Man et al. [23,24]. Lin et al. further highlighted the impact of venular narrowing in myopic eyes [25]. The modulation of cytokine expression also plays a role. Kulshrestha et al. and Hong et al. reported lower placental growth factor (PLGF) and VEGF levels in eyes with increased AL [26,27].

Longer AL has been associated with reduced retinal vascular density, potentially limiting exposure of retinal capillaries to hyperglycemia-induced damage. As the retinal surface expands with AL elongation, capillary density per area may decrease, reducing metabolic demand and inflammatory stimuli. Structural changes in highly myopic eyes, such as retinal thinning and elongation, may reduce oxygen consumption and vulnerability to hypoxia - a key driver of DR. Additionally, increased ocular volume may dilute pro-inflammatory cytokines like VEGF, attenuating angiogenic and inflammatory cascades. Alterations in retinal hemodynamics have been documented in eyes with increased AL, including reduced blood flow and perfusion pressure. These changes may lower oxidative stress and the accumulation of harmful glucose metabolites, protecting against DR-related endothelial injury [28, 29]. Ocular biomechanical properties influenced by AL may also contribute. Axial elongation causes retinal and choroidal thinning, which may redistribute mechanical stress and reduce capillary fragility. Furthermore, altered diffusion patterns in elongated eyes may reduce hyperglycemic and hypoxic burden at the cellular level [26].

The inverse association between AL and DR carries significant clinical relevance. Integrating AL into screening protocols may optimize resource use by allowing longer intervals between eye exams for patients with longer AL and closer monitoring for those with shorter AL. Incorporation of AL into risk models - alongside factors such as diabetes duration, HbA1c, and blood pressure - could enhance risk stratification and support personalized care.

Therapeutic decisions may also benefit patients with shorter AL, who may require stricter glycemic control or earlier interventions, while longer AL may justify a more conservative approach. Unraveling the mechanisms behind AL’s protective effect could reveal novel therapeutic targets. Longitudinal and mechanistic studies, especially those using advanced imaging and molecular tools, are essential.

Although this study represents the first attempt to explore the AL-DR relationship in a Greek population using a systematic methodology, some limitations must be considered. First, its cross-sectional design does not allow causal inferences. While statistically significant differences in AL were found between diabetic and non-diabetic subjects and across DR stages, causality cannot be established. Second, DR staging relied primarily on indirect ophthalmoscopy under mydriasis, complemented by OCT imaging for macular edema detection. However, fundus photography was not used, which could have enhanced reproducibility and allowed for independent verification. Third, although AL was measured using the highly accurate IOL Master 500, other biometric parameters (e.g., anterior chamber depth or lens thickness) were not assessed.

Fourth, data were collected from a single academic center, potentially limiting the generalizability of the findings. Fifth, despite strict inclusion/exclusion criteria, certain potential confounders - such as refractive error, cataract surgery history, or family history - were not accounted for. This study did not adjust for systemic confounders such as HbA1c, hypertension, and lipid profile, which may influence DR risk and act as potential confounders. The lack of standardized fundus photography may also limit reproducibility and comparability with other studies. Finally, outlier AL values due to technical measurement error were excluded from analysis to ensure data validity.

The limitations outlined above should be carefully considered when interpreting the study findings. The cross-sectional design precludes establishing causal relationships between AL and DR, meaning the observed associations should be viewed as hypothesis-generating rather than confirmatory. The lack of adjustment for systemic confounders, such as HbA1c, hypertension, and lipid profile, may have introduced residual confounding, potentially influencing both the direction and strength of the associations. Similarly, the absence of standardized fundus photography and ETDRS-based grading may reduce the reproducibility of DR classification and limit comparability with other cohorts. The single-center setting further restricts generalizability, and unmeasured ocular factors may also have influenced outcomes. Taken together, these factors underscore the need for cautious interpretation of the results and highlight the importance of future longitudinal, multicenter studies with more comprehensive systemic and ocular data collection.

Nonetheless, this study provides primary data on AL-DR relationships in a Greek population and serves as a basis for future multicenter, longitudinal investigations with comprehensive ocular biometry. Given the cross-sectional nature of this study, causality cannot be inferred, and the observed inverse association between AL and DR should be interpreted as hypothesis-generating rather than confirmatory.

## Conclusions

This study revealed a statistically significant association between AL and the presence of T2D, as well as with specific features of DR. Diabetic patients exhibited, on average, a shorter AL compared to non-diabetic individuals, a finding that may suggest a different morphological configuration of the eye influenced by the metabolic disease. These findings support the hypothesis that longer AL may be associated with a lower prevalence and severity of DR. However, this association does not establish causality, and further longitudinal studies are needed to confirm this potential protective relationship.

Although certain clinical parameters, such as diabetes duration, insulin use, or the presence of diabetic macular edema, did not show statistically significant associations with AL, the findings underscore the value of biometric assessment of the eye in the diabetic population. Overall, the exploration of AL as a potential risk biomarker for diabetic eye disease warrants further prospective and multicenter research, aiming to enhance predictive accuracy and support individualized ophthalmologic care for diabetic patients.
